# Novavax NVX-COV2373 triggers neutralization of Omicron sub-lineages

**DOI:** 10.1038/s41598-023-27698-x

**Published:** 2023-01-21

**Authors:** Jinal N. Bhiman, Simone I. Richardson, Bronwen E. Lambson, Prudence Kgagudi, Nonkululeko Mzindle, Haajira Kaldine, Carol Crowther, Glenda Gray, Linda-Gail Bekker, Novavax trial clinical lead author group, Anthonet Koen, Lee Fairlie, Leon Fouche, Qasim Bhorat, Keertan Dheda, Michele Tameris, Mduduzi Masilela, Zaheer Hoosain, Nishanta Singh, Sherika Hanley, Moherndran Archary, Cheryl Louw, Coert Grobbelaar, Umesh Lalloo, Natasha Joseph, Gertruida Kruger, Vivek Shinde, Chijioke Bennett, Gregory M. Glenn, Shabir A. Madhi, Penny L. Moore

**Affiliations:** 1 National Institute for Communicable Diseases of the National Health Laboratory Services, Johannesburg, South Africa; 2 MRC Antibody Immunity Research Unit, School of Pathology, University of the Witwatersrand, Johannesburg, South Africa; 3 The South African Medical Research Council, Tygerberg, South Africa; 4 Institute of Infectious Disease and Molecular Medicine, University of Cape Town, Cape Town, South Africa; 5 The Desmond Tutu HIV Centre, University of Cape Town, Cape Town, South Africa; 6 Novavax, Inc., Gaithersburg, MD USA; 7 South Africa Medical Research Council Vaccines and Infectious Diseases Analytics Research Unit, Faculty of Health Science, University of the Witwatersrand, Johannesburg, South Africa; 8 Centre for the AIDS Programme of Research in South Africa, University of Kwazulu-Natal, Durban, South Africa; 9 Wits Vaccines and Infectious Diseases Analytics (VIDA) Research Unit, Johannesburg, South Africa; 10 Faculty of Health Sciences, Wits RHI, University of the Witwatersrand, Johannesburg, South Africa; 11 Limpopo Clinical Research Initiative, Limpopo, South Africa; 12 Soweto Clinical Trials Centre (SCTC), Soweto, South Africa; 13 Division of Pulmonology, Department of Medicine, Centre for Lung Infection and Immunity, UCT Lung Institute and South African MRC/UCT Centre for the Study of Antimicrobial Resistance, University of Cape Town, Cape Town, South Africa; 14 Department of Immunology and Infection, Faculty of Infectious and Tropical Diseases, London School of Hygiene and Tropical Medicine, London, UK; 15 Department of Pathology, Faculty of Health Sciences, Institute of Infectious Disease and Molecular Medicine and Division of Immunology, South African Tuberculosis Vaccine Initiative (SATVI), University of Cape Town, Observatory, Cape Town, South Africa; 16 Setshaba Research Centre (SRC), Soshanguve, South Africa; 17 Josha Research, Bloemfontein, South Africa; 18 HIV and Other Infectious Diseases Research Unit (HIDRU), Verulam and Isipingo Clinical Research Site, South African Medical Research Council, Durban, South Africa; 19 Department of Family Medicine, University of KwaZulu-Natal, Durban, South Africa; 20 Durban International Clinical Research Site, Enhancing Care Foundation, Durban, South Africa; 21 Madibeng Centre for Research, Brits, South Africa; 22 Pretoria Clinical Research Centre, The Aurum Institute, Johannesburg, South Africa; 23 KwaPhila Health Solution, Cape Town, South Africa; 24 Peermed CTC (PTY) - MERC Kempton, Kempton Park, South Africa; 25 Mzansi Ethical Research Centre, Middleburg, South Africa

**Keywords:** Infectious diseases, SARS-CoV-2, Vaccines

## Abstract

The SARS-CoV-2 Omicron (B.1.1.529) Variant of Concern (VOC) and its sub-lineages (including BA.2, BA.4, BA.5, BA.2.12.1) contain spike mutations that confer high level resistance to neutralizing antibodies induced by vaccination with ancestral spike or infection with previously circulating variants. The NVX-CoV2373 vaccine, a protein nanoparticle vaccine containing the ancestral spike sequence, has value in countries with constrained cold-chain requirements. Here we report neutralizing titers following two or three doses of NVX-CoV2373. We show that after two doses, Omicron sub-lineages BA.1 and BA.4/BA.5 were resistant to neutralization by 72% (21/29) and 59% (17/29) of samples respectively. However, after a third dose of NVX-CoV2373, we observed high titers against Omicron BA.1 (GMT: 1,197) and BA.4/BA.5 (GMT: 582), with responses similar in magnitude to those triggered by three doses of an mRNA vaccine. These data are of particular relevance as BA.4/BA.5 is dominating in multiple locations, and highlight the potential utility of the NVX-CoV2373 vaccine as a booster in resource-limited environments.

## Introduction

The SARS-CoV-2 Omicron (B.1.1.529) Variant of Concern (VOC)^1^ and its sub-lineages^2,3^ (including BA.2, BA.4, BA.5, BA.2.12.1) contain changes to the spike driven by immune escape, and are relatively immune evasive compared with the ancestral-like virus to neutralizing antibodies elicited by coronavirus disease 2019 (COVID-19) vaccines^4,5^. Similarly, individuals infected with SARS-CoV-2 exhibit reduced neutralizing titers against multiple Omicron sub-lineages^4^, with BA.4 and BA.5 currently detected in over 95 countries and the latter dominating globally. While BA.4 and BA.5 have been classified as distinct sub-lineages, they share the same dominant spike mutations.

Neutralization escape by the Omicron VOC has also been observed following vaccination, regardless of the vaccine type and platform^4–9^, including with two doses of the NVX-CoV2373 vaccine where a 16-fold lower titer than the ancestral variant was observed^10^. However, booster doses, especially using mRNA vaccines, enhance neutralization capacity against Omicron^5,8^. The NVX-CoV2373 vaccine, which was tested in two phase 3 trials in the US, UK and Mexico demonstrated 90% efficacy against symptomatic and 100% efficacy against severe COVID-19, when ancestral-like and Alpha variants dominated the infections^11,12^. A Phase 2b trial in South Africa in 2020–2021 demonstrated 48% efficacy against symptomatic COVID-19, likely due to relatively antibody-evasive neutralization resistant Beta variant, despite 100% efficacy against severe disease^13^. The vaccine has received authorization for use by the European Medicines Agency, is listed on the World Health Organization’s emergency use listing for COVID-19 vaccines and has received emergency use authorization from the US FDA^13–16^. This protein-based vaccine is appealing in low-and middle-income countries (LMICs) because of its stability and reduced cold chain requirements. Here, we investigated the effect of a third dose on the neutralizing capacity of NVX-CoV2373 vaccinee sera.

## Results

**Figure 1:**
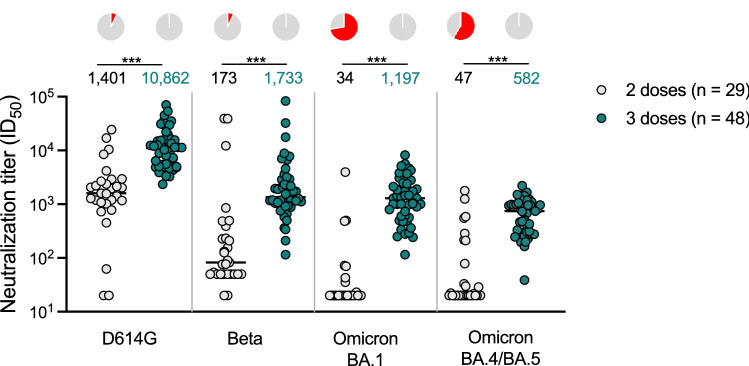
Neutralization of SARS-CoV-2 variants by NVX-CoV2373 vaccinee plasma. Neutralization of ancestral D164G, Beta, Omicron BA.1 and Omicron BA.4/BA.5 pseudoviruses by NVX-CoV2373 vaccinee plasma following 2 (grey) or 3 (teal) doses. Samples were collected 14 days after the second dose and 1 month after the third dose. Geometric mean titers (GMT) for each virus are shown above the individual points, and percent of specimens where no neutralization was observed (red) is indicated in the pie charts. Number of vaccinee specimens tested are indicated and p values were calculated using the Mann–Whitney t-test for non-parametric data with *p* < 0,001 for D614G, Beta, Omicron BA.1 and Omicron BA.4/BA.5. Samples were used at a starting dilution of 1 in 20 (limit of detection) with a seven threefold dilutions to create a titration series.

Using a spike-pseudotyped assay, we tested neutralization of the ancestral D614G, Beta, Omicron BA.1 and Omicron BA.4/BA.5 by NVX-CoV2373 vaccinee sera following a 2 dose (n = 29) and 3 dose (n = 48) regimen. These sera were collected from 52 individuals, of which 37% were female and 50% were under 29 years of age (Supplementary table 1). Fourteen days after two doses of NVX-CoV2373, geometric mean titers (GMT) were highest against the D614G variant (GMT: 1,401), with reductions in GMT to 173 (8.1-fold reduction), 34 (41-fold reduction) and 47 (30-fold reduction) against Beta, Omicron BA.1 and Omicron BA.4/BA.5 respectively. For the Omicron sub-lineages BA.1 and BA.4/BA.5, titers were lower than the limit of detection of the assay for 72% (21/29) and 59% (17/29) of samples, respectively, after the 2nd dose of vaccine (Fig. 1, grey).

At 1 month after the third dose of the NVX-CoV2372 vaccine, neutralizing antibody activity was evident against the Beta and Omicron BA.1 variants in all samples, in contrast to 2 weeks post the two doses discussed above (Fig. 1, pie charts). The neutralizing antibody titers against the D614G variant were boosted to a GMT of 10,862. Furthermore, we observed a significant ten-, 35- and 12-fold increase in titers against Beta (GMT: 1733), Omicron BA.1 (GMT: 1197) and Omicron BA.4/BA.5 (GMT: 582) respectively (Fig. 1, teal), though boosted titers were six- to 18-fold lower than those against D614G.

**Figure 2:**
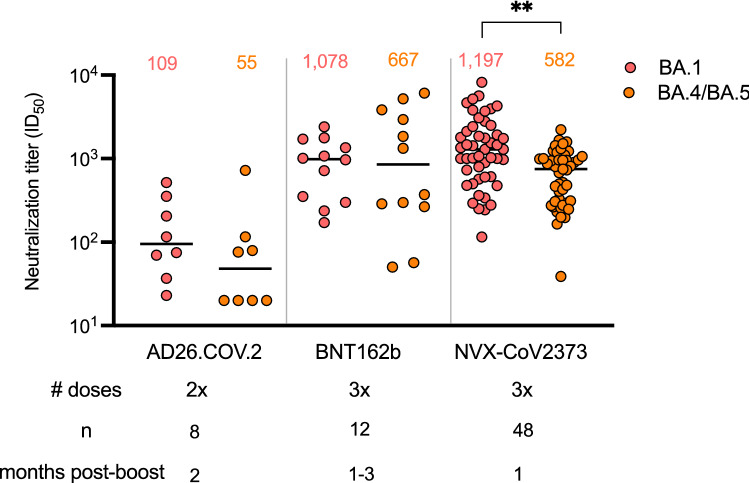
Neutralization of Omicron BA.1 and BA.4/BA.5 by boosted vaccinee plasma. Neutralization of Omicron BA.1 and BA.4/BA.5 by vaccinee plasma following 2 doses of the AD26.COV2S or 3 doses of the BNT162b2 or NVX-CoV2373 vaccines. Number of doses, number of samples and date of sample collection after boost for each group are indicated. Geometric mean titers (GMT) for each virus are shown above the individual points, *P* values were calculated using two-way ANOVA with *p* < 0.001 for AD26CoV2.S versus NXV-CoV2373 and *p* = 0.0011 for NVX-CoV2373 BA.1 versus BA.4/BA.5). Samples were used at a starting dilution of 1 in 20 (limit of detection) with a seven threefold dilutions to create a titration series.

We next compared neutralization of Omicron BA.1 and BA.4/BA.5 following multi-dose regimens of adenoviral, mRNA and protein-based vaccines. We tested samples after 2 doses of the adenoviral or 3 doses of the mRNA vaccines, given that these are currently offered as booster regimens in South Africa. These sera were collected from 11 individuals who received the AD26.COV2S vaccine (73% were female and all were over 30 years of age) and nine individuals who received the BNT162b2 vaccine (100% female and 22% were under 29 years of age, Supplementary table 1). As expected, 2 doses of AD26.COV2.S elicited ten- and 14-fold lower GMT against BA.1 than 3 doses of the BNT162b2 and NVX-CoV2373 vaccines respectively (Fig. 2). Similarly the 2 dose AD26.COV2.S vaccine elicited 12- and 11-fold lower GMT against BA.4/BA.5 than 3 does of either the BNT162b2 and NVX-CoV2373 vaccines. All third dose BNT162b2 and NVX-CoV2373 plasma were able to neutralize Omicron BA.1 and BA.4/BA.5, while only 13–50% of the two dose AD26.COV2.S samples had neutralizing activity against these Omicron sub-lineages. The NVX-CoV2373 third dose plasma GMT against BA.1 and BA.4/BA.5 was comparable to the BNT162b2 titres.

## Discussion

In summary, we report enhanced neutralization of Omicron BA.1 and BA.4/BA.5 following three doses of the NVX-CoV2373 vaccine with responses comparing well to three doses of an mRNA vaccine. We note that 6 months after two doses of NVX-CoV2373, increased binding antibodies were reported, suggesting that responses after the third dose may mature further^10^. As durability of vaccine platforms varies, future studies should assess this for NVX-CoV2373 neutralization at later time-points^10^. The two dose NVX-CoV2372 vaccine regimen elicits robust memory CD4^+^ and CD8^+^ T cell responses in 100% and 65% of individuals respectively^7,10^. In addition, the two dose regimen induces antibodies with multiple Fc-mediated functions, which in non-human primate and human cohorts likely contribute to protection from infection^10^. This T cell and Fc effector function data, which is unlikely to differ following a third dose of the NVX-CoV2373 vaccine, coupled with neutralizing antibodies we have described here, suggests that this vaccine is likely to prevent severe disease after SARS-CoV-2 breakthrough infection with Omicron BA.4/5 sub-lineages (Supplementary Table 1).

Limitations of the study include the difference in timing of the sample collection following the second and third dose of the NVX-CoV2373 vaccine, with collection at 14 days and 1 month post vaccination respectively. Similarly, sample collection following administration of the AD26COV2.S and BNT162b2 booster doses varies between 1 and 3 months and includes relatively small sample numbers. Despite these limitations, this study highlights the neutralizing titres elicited by a third dose of the NVX-CoV2373 against currently circulating Omicron sub-lineages, which supports the use of this vaccine as a booster regimen^17^ in countries where mRNA cold chain requirements cannot be met due to limited infrastructure.

## Methods

### Samples and ethics approvals

Individuals vaccinated with two or three doses of the NVX-CoV2373 vaccine were sampled at 14 days after the second dose or 35 days after the third dose. The third NVX-CoV2373 dose was administered 6 months after the first dose. This trial is registered under the ClinicalTrials.gov number, NCT04533399 (registered 17/09/2020), and the protocol was approved by the South African Health Products Regulatory Authority and by the institutional review board at each trial centre as described in detail by Shinde and colleagues^13^. Health care workers vaccinated with two dose of AD26.COV2.S (5 × 10^10^ viral particles) as part of the Sisonke implementation trial were sampled at 2 months after vaccination. This trial is registered under the ClinicalTrials.gov number, NCT05148845, and the protocol was approved by the South African Health Products Regulatory Authority. These Sisonke individuals were recruited at the National Institute for Communicable Diseases (NICD), Johannesburg. Individuals vaccinated with two and three doses of the BNT162b22 vaccine were sampled at 2 months after the second dose or 1–3 months after the third dose and were recruited from Johannesburg. This study was given ethics approval by the University of the Witwatersrand Human Research Ethics Committee (Medical) M210465. All individuals provided written informed consent and all research was performed in accordance with the relevant guidelines/regulations and in accordance with the Declaration of Helsinki.

### Lentiviral pseudovirus production and neutralization assay

The 293 T/ACE2. MF cells modified to overexpress human ACE2 were kindly provided by M. Farzan (Scripps Research). Cells were cultured in DMEM (Gibco BRL Life Technologies) containing 10% heat-inactivated fetal bovine serum (FBS) and 3 μg ml^−1^ puromycin at 37 °C, 5% CO_2_. Cell monolayers were disrupted at confluency by treatment with 0.25% trypsin in 1 mM EDTA (Gibco BRL Life Technologies). The SARS-CoV-2, Wuhan-1 spike, cloned into pCDNA3.1 was mutated using the QuikChange Lightning Site-Directed Mutagenesis kit (Agilent Technologies) to include D614G (ancestral D164G) or L18F,D80A, D215G, Δ242-244, K417N, E484K, N501Y, D614G, A701V (Beta) or Δ69-70, T915I, Δ143-145, Δ211, L212I, ins 214 EPE, G339D, S371L, S373P, S375F, K417N, N440K, G446S, S477N, T478K, Q493R, G496S, Q498R, N501Y, Y505H, T547K, D614G, N679K, P681H, N764K, D796Y, N856K, Q954H, N969K, L981F (Omicron BA.1) or T19I, L24S, Δ25–27, Δ69–70, G142D, V213G, G339D, S371F, S373P, S375F, T376A, D405N, R408S, K417N, N440K, L452R, S477N, T478K, E484A, F486V, Q498R, N501Y, Y505H, D614G, H655Y, N679K, P681H, N764K, D796Y, Q954H, N969K (Omicron BA.4/BA.5). Pseudoviruses were produced by co-transfection with a lentiviral backbone (HIV-1 pNL4.luc encoding the firefly luciferase gene) and either of the SARS-CoV-2 spike plasmids with PEIMAX (Polysciences). Culture supernatants were clarified of cells by a 0.45 μM filter and stored at − 80 °C. Plasma samples were heat-inactivated and clarified by centrifugation. Pseudovirus and serially diluted plasma/sera were incubated for 1 h at 37 °C, 5% CO_2_. Cells were added at 1 × 10^4^ cells per well after 72 h of incubation at 37 °C, 5% CO_2_, luminescence was measured using PerkinElmer Life Sciences Model Victor X luminometer. Neutralization was measured as described by a reduction in luciferase gene expression after single-round infection of 293 T/ACE2.MF cells with spike-pseudotyped viruses. Titers were calculated as the reciprocal plasma dilution (ID_50_) or monoclonal antibody concentration (IC_50_) causing 50% reduction of relative light units. Equivalency was established through participation in the SARS-CoV-2 Neutralizing Assay Concordance Survey Concordance Survey 1 run by EQAPOL and VQU, Duke Human Vaccine Institute. Cell-based neutralization assays using live virus or pseudovirus have demonstrated high concordance, with highly correlated 50% neutralization titers (Pearson r = 0.81–0.89).

## Supplementary Information

Supplementary Tables.

## Data Availability

All data reported in this paper will be shared by the lead contacts, Penny L. Moore (pennym@nicd.ac.za) and Shabir Madhi (Shabir.Madhi@wits.ac.za) upon request. This paper does not report original code.
