# Supplementary material for: Novavax NVX-COV2373 triggers neutralization of Omicron sub-lineages
Source: Sci Rep. 2023 Jan 21;13:1222. doi: 10.1038/s41598-023-27698-x (PMC9867547; doi:10.1038/s41598-023-27698-x)
Supplement: Supplementary file 1 — Supplementary Tables. [file 41598_2023_27698_MOESM1_ESM.docx]

**Supplementary information**

**Novavax NVX-COV2373 triggers neutralization of Omicron sub-lineages**

Jinal N. Bhiman^1,2^, Simone I. Richardson^1,2^, Bronwen E. Lambson^1,2^, Prudence Kgagudi^1,2^, Nonkululeko Mzindle^1,2^, Haajira Kaldine^,1,2^, Carol Crowther^1,2^, Glenda Gray^3^, Linda-Gail Bekker^4,5^, Novavax trial clinical lead author group*, Vivek Shinde^6^, Chijioke Bennett^6^, Gregory M. Glenn^6^, Shabir A. Madhi^7#^, Penny L. Moore^1,2,4,8, #^

* Novavax trial clinical lead author group* - Anthonet Koen^10^, Lee Fairlie^11^, Leon Fouche^12^, Qasim Bhorat^13^, Keertan Dheda^14,15^, Michele Tameris^16^, Mduduzi Masilela^17^, , Zaheer Hoosain^18^, Nishanta Singh^19^, Sherika Hanley^8,9^, Moherndran Archary^20^, Cheryl Louw^21^, Coert Grobbelaar^22^, Umesh Lalloo^23^, Natasha Joseph^24^, Gertruida Kruger^25^

**Affiliations**

^1^National Institute for Communicable Diseases of the National Health Laboratory Services, Johannesburg, South Africa

^2^MRC Antibody Immunity Research Unit, School of Pathology, University of the Witwatersrand, Johannesburg, South Africa

^3^The South African Medical Research Council, Tygerberg, South Africa

^4^Institute of Infectious Disease and Molecular Medicine, University of Cape Town, Cape Town, South Africa

^5^The Desmond Tutu HIV Centre, University of Cape Town, Cape Town, South Africa

^6^Novavax, Inc, Gaithersburg, Maryland, United States

^7^South Africa Medical Research Council Vaccines and Infectious Diseases Analytics Research Unit, Faculty of Health Science, University of the Witwatersrand, Johannesburg, South Africa

^8^Centre for the AIDS Programme of Research in South Africa, University of Kwazulu-Natal, Durban, South Africa

^9^Department of Family Medicine, University of KwaZulu-Natal, Durban, South Africa

^10^Wits Vaccines and Infectious Diseases Analytics (VIDA) Research Unit

^11^Wits RHI, Faculty of Health Sciences, University of the Witwatersrand, Johannesburg, South Africa.

^12^Limpopo Clinical Research Initiative, Limpopo, South Africa

^13^Soweto Clinical Trials Centre (SCTC), Soweto, South Africa

^14^ Centre for Lung Infection and Immunity, Division of Pulmonology, Department of Medicine and UCT Lung Institute & South African MRC/UCT Centre for the Study of Antimicrobial Resistance, University of Cape Town, Cape Town, South Africa.

^15^Faculty of Infectious and Tropical Diseases, Department of Immunology and Infection, London School of Hygiene and Tropical Medicine, London, UK

^16^South African Tuberculosis Vaccine Initiative (SATVI), Department of Pathology, Institute of Infectious Disease and Molecular Medicine and Division of Immunology, Faculty of Health Sciences, University of Cape Town, Observatory, Cape Town

^17^Setshaba Research Centre (SRC), South Africa

^18^Josha Research, South Africa

^19^Verulam and Isipingo Clinical Research Site, South African Medical Research Council, HIV and other Infectious Diseases Research Unit (HIDRU), South Africa

^20^Durban International Clinical Research Site, Enhancing Care Foundation, South Africa

^21^Madibeng Centre for Research, South Africa

^22^The Aurum Institute; Pretoria Clinical Research Centre, South Africa

^23^KwaPhila Health Solution, South Africa

^24^Peermed CTC (PTY) - MERC Kempton, South Africa

^25^Mzansi Ethical Research Centre Middleburg, South Africa

# Corresponding authors: email - Shabir.Madhi@wits.ac.za, [pennym@nicd.ac.za](mailto:pennym@nicd.ac.za)

**Supplementary Table 1. Vaccinee metadata**
